# Extent, consequences and economic burden of road traffic crashes in Iran

**DOI:** 10.5249/jivr.v6i2.191

**Published:** 2014-07

**Authors:** Satar Rezaei, Mohammad Arab, Behzad Karami Matin, Ali Akbari Sari

**Affiliations:** ^a^School of Public Health, Kermanshah University of Medical Sciences, Kermanshah, Iran.; ^*b*^Department of Health Management and Economics, School of Public Health, Tehran University of Medical Sciences, Tehran, Iran.

**Keywords:** Road traffic crash, Intangible cost, Economic burden

## Abstract

**Background:**

Road Traffic Injuries (RTIs) as a result of road traffic crashes (RTCs) rank as the leading cause of death, disability and property loss worldwide, especially in low and middle-income countries. This study aims to analyze the costs of RTCs in Iran.

**Methods:**

A standard human capital approach was used to estimate the costs. Costs included medical, administrative and funeral costs, property damage, production lost and intangible costs. Data about the number of deaths and injuries resulting from RTIs between 20 March 2009 and 20 March 2010 was obtained from two national databases designed at the Center for Disaster Management and Medical Emergencies (CDMME) and the Legal Medicine Organization (LMO), respectively. The severity and medical costs of injuries were identified by reviewing 400 medical records that were selected randomly from patients who were admitted to two large trauma centers in Shariati and Sina hospitals in Tehran province. Moreover, information about production lost, property damage, rehabilitation cost, intangible costs and administration costs were collected by review of current evidence and consulting with expert opinion.

**Results:**

In total 806,922 RTIs and 22,974 deaths resulted from the RTCs in the study period. The total cost of RTCs was about 72,465 billion Rials (7.2 billion US Dollars), which amounts to 2.19% of Iran’s Gross Domestic Production (GDP). Direct costs were 3,516 billion Rials (around 48.6 % of the total costs), following by 24,785 billion Rials (around 34.2 % of the total costs) for production lost and 12,513 billion Rials (around 17.2 % of the total costs) for intangible costs.

**Conclusions:**

This study indicated that the burden of both RTCs and RTIs in Iran is substantial. Moreover, RTCs have significant economic consequences and are a large drain on healthcare resources.

## Introduction

As a major public health problem in recent years, injuries account approximately for 10% of the total cost of diseases worldwide. In low and middle-income countries (LMICs) they are responsible for around 90% of injury-related mortalities.^1^ The number of injuries and thus their cost can be evaluated by different methods including hospital and death records,^2^ relying on register systems as well as population-based studies that are usually expensive.^3^ Among them road traffic accidents is one of the most leading causes of injuries, which approximately cover 25% of all injuries in the world.^1^

Road traffic crashes (RTCs) and thus road traffic injuries (RTIs) are among the leading causes of death, disability and property loss worldwide.^4^ Each year approximately 1.2 million people die and an extra 20 to 50 million people are injured or become disabled from the RTCs in the world.^1^ RTCs are responsible for approximately 23% of deaths due to injuries, and about 90% of fatal RTIs occur in the LMICs.^1^ Recent studies have shown an increasing trend of fatalities caused by RTIs in several LMICs including Pakistan, Bangladesh and Nepal.^5-8^

The UK Transport Research Laboratory Report, published in 2000, estimated that the global cost of RTCs was around 518 billion US dollars ($US) annually, and 65 billion dollars of these are attributable to LMICs, which is more than the financial support they normally receive from high-income countries.^1,9^ Focusing on the cost of injury by means of gross national product (GNP) and based on a WHO report on RTIs, RTIs’ cost approximately is 1% of GNP in low-income countries, and around 1.5% in middle-income countries, followed by 2% in high-income countries.^1^

In Iran RTIs is the second largest cause of death after cardiovascular diseases.^10,11^ Several studies have been conducted in Iran, but the main focus has been on the epidemiological pattern or advocacy of RTIs as well as post-crash events.^1^ One study in Iran showed that they account for around 5% of the GDP, which is actually more than the GDP of some countries. In recent years the Iranian government has decided to reduce the number of fatal RTIs by 50% in the whole country,^2,12^ by means of a new national policy. Evaluating the extent, environmental costs and consequences of RTCs is an important step in planning for their prevention and reducing their costs. Moreover, WHO emphasize that all activities for prevention of RTIs, should be based on valid and reliable evidence. To our best knowledge there are limited studies about the cost of RTIs in Iran. Accordingly the aim of this study is to analyze these costs using a standard human capital (HC) approach.

## Methods

This is a cross sectional study carried out between 20 March 2009 and 20 March 2010. The Human Capital approach is one of the main methods for estimating the costs of RTCs.^13^ In the HC approach the cost of RTCs is estimated as the sum of resource costs (i.e. property damage, medical and police costs and production lost) and other costs normally named as intangible costs (pain, grief and suffering).^13^ In this study a standard human capital approach was used^14-17^ to estimate the costs of RTCs in Iran. 

In this study the costs were divided into three domains: direct, indirect and intangible costs. Direct costs included pre-hospital, hospital, post-hospital (physical therapy and rehabilitation), administrative and funeral costs and the cost of property damages. Indirect costs were considered as production lost. Finally intangible costs were considered as cost of pain, grief and suffering. The costs were estimated according to the severity and the level of injuries resulted from the RTCs. All the costs were calculated in Iranian Rials (IRR). At the time of the study, the exchange rate for a US Dollar was about 10.000 IRR.

The severity of injuries was classified into four groups including fatal injury, serious injury, slight injury and property damage only.^13-15^ The medical costs, administrative costs, funeral costs, intangible costs and production lost were put in one category of costs and these costs have calculated by the severity of injuries. Finally the costs of property damage were calculated separately.

According to current practice, for all the cases that are transferred to hospitals after RTIs, information should be reported to the CDMME every month. Therefore, we considered the CDMME database as the best available database for the number of RTIs. On the other hand, for all the cases of fatal RTIs, information is normally reported to the Legal Medicine Organization (LMO) database irrespective of whether death occurs at the RTC scene, at the pre-hospital phase or at hospital. Therefore, the LMO database was considered as the best available database for the number of fatal RTIs. The data about the number of RTCs and number of people that were injured in these accidents were collected from the database specially designed at the CDMME, Ministry of Health and Medical Education (MOHME).^18^ Data about the number of fatalities due to RTIs were obtained from the database specially designed at Iran LMO.^19^

**Pre-hospital costs**

53% of cases that are injured in RTC are transferred to the hospitals by the Emergency Medical Services (EMS) and the remaining 47% make their own way to hospitals or are transferred by members of the public like taxi drivers, commercial drivers, police officers or laypeople. In addition, around 24% of patients that are transported to hospital by the EMS are involved in RTCs;^18^ therefore we estimated that 24% of the annual EMS budget was used to transport injured people from the RTC scene. We calculated that the remaining injured people made their own way to the hospital or by other means and estimated 10 US Dollars for each of these transportations. 

**Hospital costs**

Since studies in Iran have shown that 38% of deaths from fatal RTIs (8,730 people) occur at hospitals and the remaining 62% occur at the scene or during transportation to the hospital. In addition, 2% (16,137) of RTIs are minor injuries and treated at the accident scene and hence do not need any transfer to hospital.^18^ Therefore the total number of RTIs referred to hospitals was estimated as 799,514 people (total injuries minus 16,137 plus 8,730).

According to the current practice in Iran, any medical procedure has a relative weight or relative value unit (RVU) that ranges between 0 and 200. These tariffs are the basis for calculating the medical costs in Iranian hospitals. In order to identify the proportion of patients with slight (length of stay less than 24 hours) and serious (length of stay more than 24 hours) RTIs and the average hospital costs related to them, medical records of 400 patients admitted to two large trauma centers (Shariati and Sina hospitals in Tehran) were reviewed in the study period. 

**Physiotherapy and rehabilitation costs**

Recent studies have shown that about half of the RTIs are orthopedic cases and 10% of them (5% of all injuries) need physiotherapy and rehabilitation services.^1^ The average cost of rehabilitation for each person was 45,000 IRR. This figure was estimated according to the tariff that is normally used for these services in Iran.

**Property damage costs**

a) Damage to vehicles: A recent study from 2004 was used to estimate the number and type of vehicles involved in the RTCs and the proportion and amount of damage to the vehicles. This study used the national insurance data and estimated that on average 18.7% of the value of each vehicle incurred damaged in the RTCs.^21^ According to inflation rate in the average price of vehicles, we added an annual rate of 10% to estimate the average price of each type of vehicle in 2009.^20^ According to this report, in each RTC on average, 1.89 vehicles were involved, therefore it was estimated that a total of 1,492,355 vehicles had been damaged in RTCs in 2009.^21^ The total cost of vehicle damages was estimated by multiplying the number of vehicles involved in RTCs by the average damage per vehicle (18.7% of the vehicle cost).

b) Damage to public property, fixed objects and reparation costs: 5% of the total vehicle costs was considered as the cost of damage to public property and fixed objects.^20^

**Administrative costs**

Administrative costs from RTCs are incurred by the police, the insurance companies and the legal services. These costs were estimated according to the Transportation Research Laboratory (TRL) as 0.2% of fatal RTI costs, 0.4% of serious injury costs, 14% of minor injury costs and 10% of property damage only.^13^

**Funeral costs**

In order to calculate this, 100 students at the Tehran University of Medical Sciences from 25 provinces of the country were asked to estimate the average cost of a funeral. The average cost of a funeral in the 25 provinces was estimated and used in this study. 

**Production lost**

Production lost was calculated for fatal, serious and minor injuries by the following formula.^15^

**Production lost of fatal injuries= [Ʃw (1+g)^i^ / (1+r)^i^]**

Where:

W=average annual GDP per capita, r=discount rate (5%), g=growth rate of the economy (3.7%, mean economic growth in the past 30 years), i=average number of years lost per crash (72-35=37 years). 

A recent study has shown that the average age of people who died in RTIs was 35 years^22^ and their life expectancy was 72 years.^23^ Therefore the average number of years lost by each fatality due to RTI was 37 years. According to the World Bank database, the average GDP per capita was 0.045 billion IRR.^24^ For serious and slight RTIs the production lost was 15 and 3 days, respectively and the wage lost was 87,000 IRR per day according to the wages in 2009.^25^

**Intangible costs**

The Asian Development Bank (ADB) recommendation was used to estimate the intangible costs as 28% of the total cost of fatal injuries, 50% of the total cost of serious injuries, 8% of the total costs of minor injuries and 0% for property damage only.^26^

## Results

Table 1 shows the majority of data including the number of injuries, total number of patients transported by EMS to hospital and number of deaths which were obtained from the CDMME, EMS and LMO databases respectively. In addition the data on damage to property, administration and intangible costs were collected from other studies. The remaining data were estimated from other sources as presented in Table 1. Overall a total of 806,922 people were injured and an additional 22,974 people died as a result of RTCs in Iran during the study period. Table 2 presents the total cost of RTCs based on cost and source of data. The total cost of RTCs was 72,465 billion Rials (Table 2). The total production lost was about 24,785 billion Rials and 98.5% of the production lost was related to fatal RTIs. Property damage and production lost were responsible for about 39.6% and 34.2% of the total RTC costs, respectively (Table 2).

**Table 1:** Types and sources of data used in estimating cost of RTCs in Iran (20 March 2009 till 20 March 2010).

Type of data	Number in 2009	Source of data
RTIs	806,922	CDMME
Deaths due to RTCs	22,974	Legal Medicine Organization(LMO)
Total patients transported by EMS to hospitals	1,817,363	EMS database
Patients transported to hospitals from the crash scene by EMS	427,559	EMS database
Total patients transported to the hospital by their own or other means	388,593	EMS database
Annual budget of the EMS	-	Accounting office at MOHME
Hospitalization due to RTIs	799,514	CDMME
The average hospital costs of each inpatient due to RTIs (IR Rials)	0.005	Review of 400 medical records in two trauma centers
The average hospital costs of each outpatient due to RTIs (billion Rials)	0.001	Review of 400 medical records in two trauma centers
Injured patients requiring physiotherapy and rehabilitation	39,537	WHO & WB report (2004)
Average price of each type of vehicles involved in RTCs	-	Ayati 2004
The total number of RTCs	789,606	www.rmto.com
Vehicles involved in RTCs	1,492,355	Ayati 2004
Average damage per vehicle	18.7 %	Ayati 2004
Production lost due to fatality	-	Equation (Anh, 2005), data of World Bank and Central Bank of Iran.
Length of hospital stay for inpatients and outpatients	-	Review of 400 medical records in two trauma centers
Wage rate in 2009 (billion Rials)	0.000087	www.irimlsa.ir
GDP per capita (billion Rials)	0.045	http://data.worldbank.org
Growth rate of the economy	3.7%	Mean economic growth in the past 30 years, www.cbi.ir
The average age of fatality (in years) of both sexes	35	Soori and et al,2009
Life expectancy (in years) for both sexes	72	www.who.org
The proportion of RTC-injured patients not attending hospitals	2%	http://ems115.behdasht.gov.ir/
Administrative costs	-	Transportation Research Laboratory (TRL),1995
Intangible costs	-	The Asian Development Bank (ADB),1997
Funeral costs	-	Perception of 100 university students (interview)

**Table 2:** The total cost of RTCs divided according to cost in Iran (20 March 2009 till 20 March 2010).

Type of costs	Cost(billion IR Rials)	Percent (%)
**Direct costs:**		
Medical care	2,952.3	4.08
Property damage	28,708.9	39.62
Administration	3,390.7	4.67
Funeral	114.8	0.16
**Indirect cost**		
Production lost	24,785	34.2
**Intangible (pain, grief and suffering)**	12,513.7	17.27
**Total costs **	**72,465.7**	**100**

The total cost of property damage was about 28,708 billion Rials, and 1,367 billion Rials (5%) of this was related to public property and fixed object damage. The total cost of vehicle damage was about 27,341 billion Rials. Medical costs from RTIs were 2,952 billion Rials (about 4.08% of the total costs), of which 2,574 billion Rials (87%) were related to hospital costs, 360.4 billion Rials (12.3%) were related to pre-hospital costs and 17.7 billion Rials (0.7%) were related to rehabilitation costs. The average cost of each fatal RTI was about 1.47 billion Rials, followed by 0.021, 0.013 and 00.02 billion Rials for property damage, serious and minor RTIs, respectively. 

The average production lost from a fatal RTI was 1.05 billion Rials, followed by 0.001 and 0.0002 billion Rials for serious and minor RTIs, respectively. The average administration costs per death, serious, minor injury and property damage only were 0.002, 0.000005, 0.00003 and 0.002 billion Rials; and the average intangible costs per death, serious and minor injuries were 0.412, 0.006 and 0.0001billion Rials, respectively (Table 3).

**Table 3:** The total costs of RTCs according to severity of injury and type of costs in Iran (20 March 2009 till 20 March 2010).

Type of costs(billion Rials) Severity of injury	Medical care	Production lost	Administration	Intangible	Funeral	Vehicle damage	Damage to public property and fixed objects	Total
Fatal injury *	47.5	24,124.6	67.6	9,471.2	114.8	-	-	33,825.9
Serious injury *	2,388.5	567.5	23.8	2,979.9	-	-	-	5,959.9
Slight injury *	516.3	92.8	109.3	62.4	-	-	-	780.9
Property damages only (for all accidents)*	-	-	3,189.8	-	-	27,341.8	1,367	31,898.8
Total costs	2,952.3	24,785	3,390.7	12,513.7	114.8	27,341.8	1,367	72,465.7

* Cost of fatal, serious and slight injury accidents excludes the cost of property damages.

## Discussion

This study, the first of its kind in Iran, indicated that the total cost of RTCs was 72,465 billion Rials in one Iranian calendar year, 48.5% of which were direct costs (medical costs, property damages, administration and funeral costs), 34.2% were related to production lost and 17.3% were intangible costs, employing HC approach. The choice of the HC approach normally depends on the research aim and data availability. Due to the lack of precise data in LMICs, the majority of studies conducted in countries like Egypt, Vietnam, Taiwan, the Philippines and Jordan have used the HC approach to estimate the cost of RTCs.^13-17^ We also used the HC approach to estimate the cost of RTCs in Iran for the first time. 

According to a report by the World Health Organization, the annual cost of RTCs in LMICs is between 1 and 2 % of GDP.^1^ The cost of RTCs in Egypt in 2008 was around 10 billion Egyptian Pounds, and about 1% of their GDP.^14^ Moreover, the cost of RTCs in Vietnam was around 0.45% of their GDP in 2004.^15^ However, the total cost of RTCs in Iran was 2.19% of GDP which is considerably more than the other countries. This shows that RTCs are a relatively large drain on the public resources in Iran compared to other countries in the region.

The intangible costs (pain, grief and suffering) and administration costs of RTCs were about 17.3% and 4.7% of the total costs, respectively. This is similar to recent studies published in Egypt^14^ and the Philippines.^16^ Our findings indicated that the production lost related to RTCs was about 24,785 billion Rials (34.2% of the total cost), which is similar to other studies.^14-16^ This seems to be more likely to be related to the mean length of hospital stay for inpatients that was 6 days and similar to the figures reported by a study from Bangladesh (5.7 days). However, it is significantly lower than the 21.5 days reported by a study that was conducted in Bangkok.^27^ This might be because we did not follow up those patients that were referred to tertiary care for further treatment. In addition, the latter study included hospitalization days, rehabilitation and recovery days. The other reasons for the difference in length of hospital stay in this study compared with other countries might be due to difference in health care delivery systems and differences in the design of the studies. However, the differences in the costs of RTCs among the countries might be partly because of variations in their health care systems, service costs, pattern of injuries, the safety of vehicles and roads, the driving culture and a variety of other factors. It is therefore, important to identify the main factors contributing to the RTCs, to develop and implement strategies to reduce their occurrence and to mitigate their consequences. 

**Limitations and strengths**

Although this study provides a valuable insight about the cost of RTCs in Iran, there are several limitations that should be considered in any future study. Using two different sources of data to estimate the number of deaths and injuries resulting from RTCs might be a limitation of this study. However we selected these databases, in particular the CDMME database, as they provide a more complete and accessible database for the number of injuries. Moreover another study has indicated that the LMO database has a better coverage and accuracy in fatal RTI registration. However, register databases usually suffer from underreporting and thus under-estimation, so we recommend caution when making interpretations.

Moreover, some data about property damage, intangible costs, administration costs, the average age of fatal RTIs, physiotherapy costs, the average cost of transportation by EMS and the mean age of fatal RTIs were not available, so we had to use data from other studies and expert opinion to estimate the costs of RTCs. Therefore, some of the costs that were estimated in this study might differ to some extent from the real costs.

## Conclusion

This study indicated that the economic burden of RTCs in Iran is substantial and the cost imposed by RTCs as a proportion of GDP in Iran is greater. While data required for estimating the real cost of RTCs may need some of surveys on national level for increased precision in estimating the real costs of RTCs; however, the estimated cost of RTCs, at least rough data is available and on national level, this is useful for analysis and policy making in improving road safety and reduction cost of RTCs. This suggests that prevention of RTCs should be considered as a top priority for the policy makers and efforts should be made to identify and prioritize improvement strategies followed up by an evaluation of their effects. 
